# Genetic and Pomological Determination of the Trueness-to-Type of Sweet Cherry Cultivars in the German National Fruit Genebank

**DOI:** 10.3390/plants12010205

**Published:** 2023-01-03

**Authors:** Stefanie Reim, Juliane Schiffler, Annette Braun-Lüllemann, Mirko Schuster, Henryk Flachowsky, Monika Höfer

**Affiliations:** 1 Julius Kühn-Institut (JKI), Federal Research Centre for Cultivated Plants, Institute for Breeding Research on Fruit Crops, Pillnitzer Platz 3a, 01326 Dresden, Germany; 2 Pomologen-Verein e.V., An der Kirche 5, 37318 Hohengandern, Germany

**Keywords:** *Prunus avium* L., genetic resources, genetic structure, parentage analysis, true-to-type, cultivar identity

## Abstract

Genebank collections preserve many old cultivars with ancient breeding history. However, often, cultivars with synonymous or incorrect names are maintained in multiple collections. Therefore, pomological and genetic characterization is an essential prerequisite for confirming trueness-to-type of cultivars in gene bank collections. In our study, 1442 single sweet cherry (*Prunus avium* L.) trees of the German Fruit Genebank were evaluated according to their trueness-to-type. For this purpose, pomological analysis was performed, in which the accessions were assigned totheir historical cultivar names. The pomological identifications were based on several historical reference sources, such as fruit references from historical cherry cultivar and fruit-stone collections, as well as historical pomological literature sources. In addition, the cherry trees were genetically analyzed for cultivar identity using 16 SSR markers. Based on pomological characterization and genetic analysis for the majority of the trees (86%), cultivar authenticity could be confirmed. Most markers were highly discriminating and powerful for cultivar identification. The cherry collection showed a high degree of genetic diversity, with an expected heterozygosity *He* = 0.67. Generally, high genetic admixture between cultivars of different geographic origin and year of origin was obtained after STRUCTURE analysis, demonstrating the extensive exchange of genetic information between cherry cultivars in the collection over time. However, the phylogenetic tree calculated by DARwin reflected the geographic origin of selected cherry cultivars. After parentage analysis with CERVUS, paternity could not be confirmed for three cultivars, indicating the necessity of further pedigree analysis for these cultivars. The results of our study underlined the general importance of evaluating the authenticity of cultivars in genebank collections based on genetic and pomological characterization.

## 1. Introduction

Sweet cherry (*Prunus avium* L.) is an economically important fruit crop that has been widely cultivated in Europe for hundreds of years. *P. avium* is usually diploid with 2n = 2x = 16 chromosomes, but sometimes triploid and tetraploid individuals occur naturally in wild cherry [1]. Like most Rosacea species, cherry is self-incompatible and needs other trees for pollination. Cherry is thought to be native to Asia Minor in the area between the Black Sea and the Caspian Sea [2,3]. The species *P. avium* includes both large-fruited domesticated sweet cherries and small-fruited wild forest so-called mazzard cherries used for timber production. Sweet cherry cultivars are commonly divided into the soft-fleshed heart cherries and the firm-fleshed Bigarreau cherries [4]. Both groups are further subdivided, although classification into distinct groups is often difficult [5].

In Germany, a large number of traditional sweet cherry cultivars were grown in the past, which are well adapted to the environmental conditions of different regions. Until the first half of the 20th century, these very different cherry cultivars were grown on a large scale on strong-growing rootstocks in orchard meadows, or for self-sufficiency in private gardens. In the second half of the 20th century and up to today, there was a significant change in cherry production, which led to large and very specialized commercial farms [6]. Intensive production systems based on a very limited number of well-selected and highly productive cultivars emerged. These few international cultivars are widely grown in most cherry-producing countries, while traditional, locally-adapted cultivars are threatened with extinction. This trend in cherry cultivation increases the risk that a significant part of the remaining diversity of cherry cultivars will be lost, which is why efforts to preserve cherry genetic resources are needed.

For this reason, the German Fruit Genebank was established in 2007 by the Federal Minister of Food and Agriculture [7,8,9]. The German Fruit Genebank is a national decentralized genebank network for fruit genetic resources (FGR), centrally coordinated by the Julius Kühn-Institut (JKI), Institute for Breeding Research on Fruit Crops in Dresden-Pillnitz, Germany. This network was established to ensure effective and long-term conservation of fruit genetic resources and to ensure their availability for research and breeding. In this context, the conservation of fruit genetic resources is carried out by various stakeholders, including federal and state research institutions and universities, nurseries, non-governmental organizations (NGOs), non-profit associations, municipalities, counties, and private individuals. The cherry network currently consists of eleven partners, where the different sweet and/or sour cherry genotypes are generally preserved *on farm* or *ex situ* in field collections.

The cherry cultivars in the various collections come from different sources without proven cultivar identity. In addition, accessions with probably synonymous names may occur and over time, a cultivar name in the collection may no longer be representative of that cultivar. Therefore, great attention is devoted to the cultivar authenticity of the preserved accessions in gene bank collections.

Since the foundation of the German Fruit Genebank, four projects on pomological and molecular determination of cultivar authenticity have been carried out in the cherry network on behalf of the Federal Ministry of Food and Agriculture. Based on the results, the trueness-to-type of the cultivars in the six genebank collections investigated was assessed. The pomological characterizations were performed by two external experts, preferably members of Pomological Commission of the German Pomological Society, with excellent knowledge in phenotypic identification of cherry cultivars. A total of 16 Simple Sequence Repeat (SSR) markers proposed by the European Cooperative Program for Plant Genetic Resources (ECPGR; [10]), as standard SSR marker set, were used for genotyping. The usage of this standard SSR marker set facilitated the comparison of SSR data between analyses from different institutions, countries, or years. Based on the pomological and genotypic characterization, the cultivar authenticity of each tree was confirmed or rejected. Genetic diversity and genetic structure analyses were performed for all confirmed true-to-type cherry cultivars, as were paternity analyses for selected cultivars. These studies are essential for the development of an appropriate genebank management strategy. Identification of duplicates, mislabeled genotypes, synonyms, and homonyms will help in efficient and sustainable management of the collections. Eliminating redundancies increases cost efficiency for existing collections and enables the inclusion of new cultivars or accessions not previously analyzed for cultivar authenticity.

## 2. Results

### 2.1. Characterization of Trueness-to-Type by Pomological and Molecular Characterization

A total of 1,442 sweet cherry trees were examined for their cultivar authenticity based on pomological and molecular characterization. By performing the pomological and molecular characterizations twice, on the one hand, ambiguities from the first examination could be clarified and, on the other hand, the pomologists could consider the molecular analyses in their evaluations. According to the workflow for the assessment of cultivar authenticity of cherries, a final determination of cultivar authenticity was made based on phenotypic and genetic data. Molecular analyses of the 1442 trees using the 16 SSR markers and pomological determination resulted in 383 unique genotypes, which were used for the subsequent genetic analyses. The genetic data of the 383 unique sweet cherry genotypes/cultivars were published in Openagrar (dataset available: https://www.openagrar.de/receive/openagrar_mods_00066782, accessed on 22 March 2021) [11]. With 1235 trees, the majority (86%) could be assigned an unambiguous cultivar name (Table 1). These trees correspond to 292 true-to-type cultivars [11].

An additional 120 trees, including 55 genotypes, were grouped into their own molecular group, but no reference cultivars were available for the pomological cultivar identification. These were mainly newer international cultivars that were included into the collection either for comparative trials or for breeding work. In addition, old local cultivars, which were pomologically interesting, had partial or no references because not all old cultivars had been described pomologically. For 64 trees, (corresponding to 21 genotypes) a pomological determination was not possible due to lack of fruit quality or quantity, and for 23 trees (corresponding to 15 genotypes), the pomological determination was approved with reservation.

The category “no pomological determination” included trees that were not examined because the trees were dying, died, or did not bear fruits during the study period. The category “pomological determination approved with reservation” included the trees bearing, in the years of investigation, insufficient amount of fruits or partially uncharacteristic fruits (especially young trees), which meant that they showed slight differences with fruits of reference trees (in one or more characteristics).

**Figure 1:**
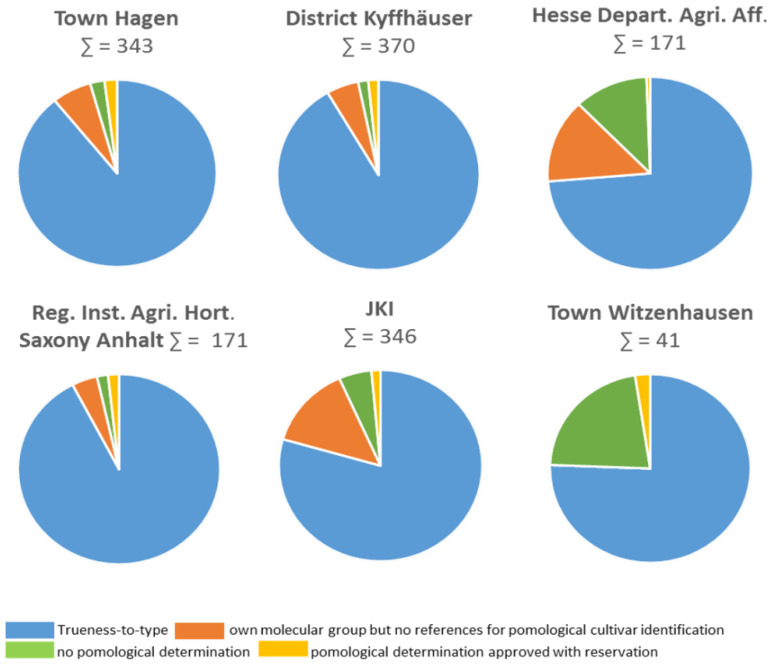
Determination of trueness-to-type based on pomological and molecular analysis data of the sweet cherry accessions of the German National Fruit Genebank (Deutsche Genbank Obst, DGO), depending on the collection of the six partners involved.

In the individual partner collections, 74–92% of the trees were determined as true-to-type. The respective proportion of the categories in the individual collections of the partners is shown in Figure 1.

### 2.2. Discrimination Power of the SSRs and Genetic Diversity

All 16 SSR markers showed clear polymorphisms and reproducible results, with one or two amplified fragments per genotype. High genetic diversity in the cherry collection was observed, with an average number of different alleles of *Na* = 15.19, ranging from *Na* = 5 for EMPA003 to *Na* = 24 for PceGA34 (Table 2). The average effective number of alleles was *Ne* = 3.82. The highest number of effective alleles was calculated for PceGA34 with *Ne* = 7.47 and the lowest number of effective alleles with *Ne* = 1.41 for EMPA017. The mean expected heterozygosity was *He* = 0.67, with the lowest value for EMPA003 (*He* = 0.38) and the highest value for EMPaS06 and PceGA34 (*He* = 0.87). The allelic richness (*Ar*) was also high for most SSR markers and ranged between *Ar* = 3.11 (EMPA003) and *Ar* = 16.27 (UDP98-412).

**Figure 2:**
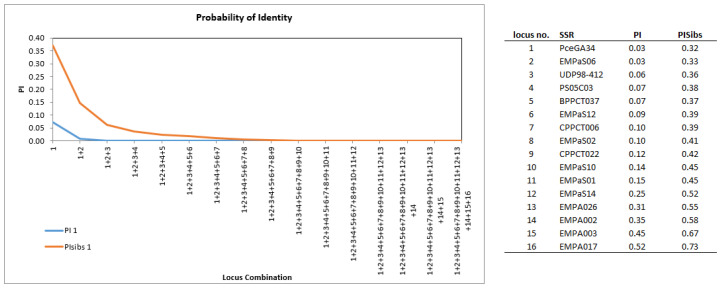
The probability of identity (*PI*) and the *PI_sib_* (taking into account related individuals in the cherry collection), based on the diminished *PI* value of each locus. Evaluation based on the analysis of 383 unique sweet cherry genotypes using 16 SSR. *PI =* Probability of Identity (2 * [Sum *(pi*^2)^2]-Sum(pi)^4); *PI_sibs_* = Probability of Identity for Sibs at a Locus = 0.25 + [0.5 * Sum(pi^2) ] + [0.5 * Sum(pi^2)^2] − [0.25 * Sum(pi)^4].

The probability of identity was low for most SSRs (mean *PI* = 0.18) and ranged from *PI* = 0.52 (EMPA017) to *PI* = 0.03 (PceGA34 and EMPaS06), indicating a high discrimination power of the used SSR markers (Figure 2). Taking into account related individuals in the cherry collection, the mean *PI_sib_* was slightly lower at 0.46. Because low *PI* values indicate high marker efficiency, the best markers were PceGA34 and EMPaS06 (both *PI* = 0.03), UDP98-412 (*PI* = 0.06), PS05C03, BPPCT037 (both *PI* = 0.07), and EMPaS12 (*PI* = 0.09). The combination of these six SSR markers were sufficient to discriminate all genotypes in the cherry collection (*PI* = 0.0). Assuming the existence of related cultivars in the cherry collection, 14 SSR markers were necessary to exclude individuals with the same multilocus genotype that may have been detected by chance.

### 2.3. Genetic Structure within the Unique Cherry Cultivars

**Figure 3:**
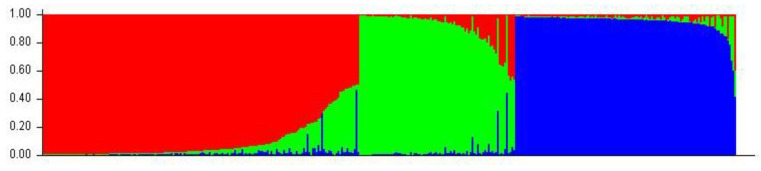
Genetic structure among the 383 cherry genotypes/cultivars based on 16 SSR markers. Each genotype is represented by a vertical bar partitioned into three genetic clusters (*K* = 3). Each color represents the estimated membership fraction of the three genetic clusters (cluster 1 = red; cluster 2 = green, cluster 3 = blue).

Following STRUCTURE analysis, 383 genotypes were grouped into three genetic clusters (*K* = 3) (Figure 3). Cluster 1 contained 174 cherry cultivars, while 123 were grouped in cluster 2 and the remaining 86 genotypes belong to the genetic cluster 3. A main part (86%) showed a clear affiliation (*Q* > 80%) to their respective genetic cluster, with low genetic admixture with the other clusters. This result suggested a distinct genetic difference between the three genetic clusters obtained in the cherry collection. However, the pairwise *F*_st_ values between the genetic clusters were 0.024 (cluster 1 and 2), 0.027 (cluster 2 and 3) to 0.029 (cluster 1 and 3) indicating only slight genetic differentiation between the single clusters [12].

**Figure 4:**
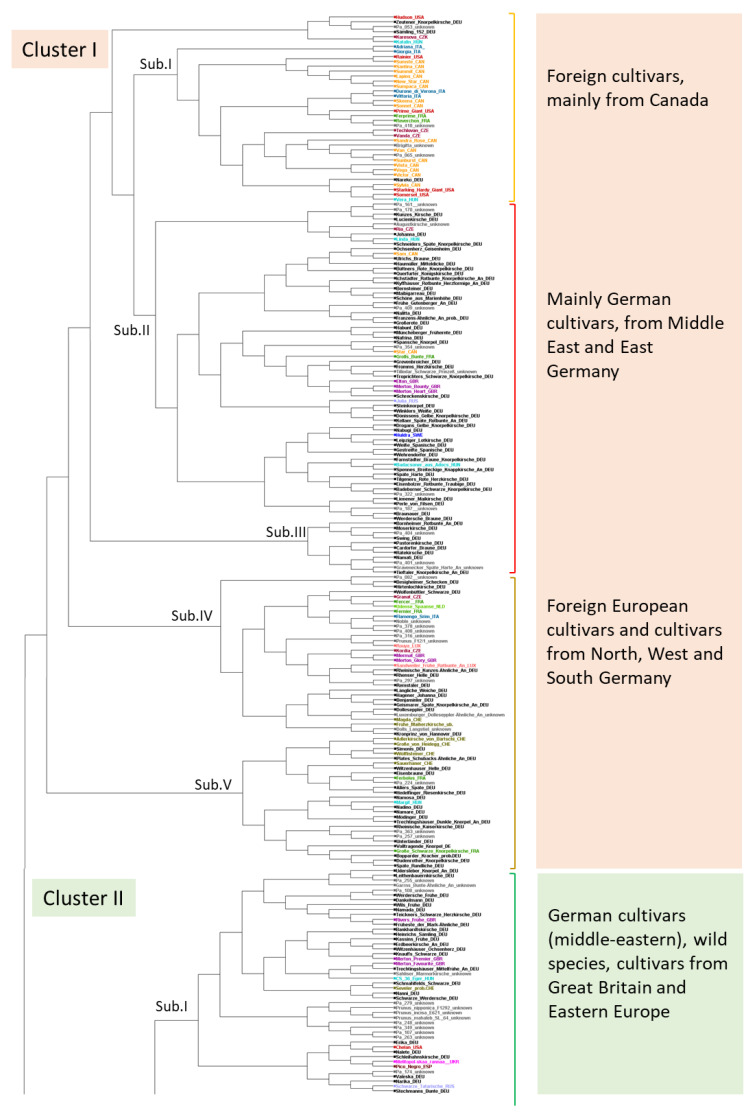

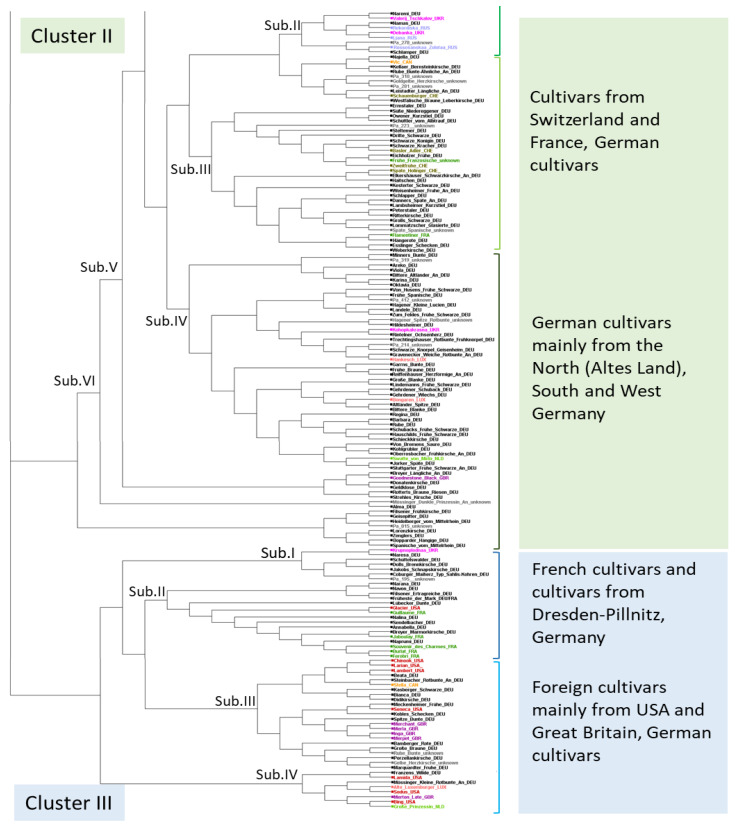
Phylogenetic relationships among sweet cherry cultivars from the German cherry genebank, based on genetic data of 16 SSR markers. The phylogenetic tree is estimated based on the dissimilarity distance matrix of the SSR data of the 383 genotypes, considering a bootstrap analysis with 1000 replications. The cherry cultivars are labeled according to their country of origin, as follows: black: Germany (DEU); yellow: Canada (CAN); red: USA (USA); green: France (FRA); purple: Great Britain (GBR); rosa: Luxembourg (LUX); bright green: The Netherlands (NLD); pink: Ukraine (UKR); olive: Switzerland (CHE); lavender: Russia (RUS); bright blue: Hungary (HUN); blue: Sweden (SWD); grey-blue: Italy (ITA); dark red: Czechia (CZE); gray: unknown origin.

Furthermore, a phylogenetic analysis using DARwin was performed to determine the genetic relationship between the single cherry cultivars. Similar to the STRUCTURE output, the dendrogram divided the 383 selected genotypes into three main clusters (Figure 4 and S1).

Cluster I was separated into five sub-clusters (cluster I, Sub.I to Sub.V). Sub.I included mainly cherry cultivars that originated from Canada, such as ‘Sumste’, ’Sunburst’, and ‘Lapins’. Furthermore, single cherry cultivars in these clusters originated from the USA (e.g., ‘Rainier’, or ‘Prime Giant’), from Italy (e.g., ‘Adriana’, ‘Vittoria’) from Czechia (e.g., ‘Vanda’) or from France (e.g., ‘Reverchon’). In contrast, Sub.II and III. included mainly German cultivars originating from the middle eastern and eastern parts of Germany (e.g., ‘Badeborner Schwarze Knorpelkirsche’; ‘Gestreifte Spanische’). Particularly, ancient cultivars from Saxony–Anhalt and Brandenburg were part of this branch. Sub.IV and V included cherry cultivars that originated from other European countries, but also German cultivars from the northern, western, and southern parts of Germany (e.g., ‘Kronprinz von Hannover’, ‘Rhenser Helle’, ‘Dolleseppler’).

Cluster II was separated into six sub-clusters (cluster II, Sub.I to Sub.VI). Sub.I and II contained cherry cultivars from Great Britain, Hungary, Russia, and the Ukraine, as well as the three wild *Prunus* species accessions (*P. incisa*, *P. mahaleb*, *P. nipponica*) that were used as ECPGR reference genotypes. In Sub.III, cultivars from Switzerland and France were grouped together with German cultivars. Sub.IV, V and VI included mostly German cultivars from different regions and only a few foreign cherry cultivars. Especially in Sub. IV, old varieties of the cherry growing region Altes Land were combined with their breeding products of the Fruit Research Station in Jork, near Hamburg. In contrast, only cultivars of the middle Rhine region were included in Sub.VI.

Cluster III was separated into four sub-clusters. Sub.I and Sub.II included mainly French cultivars (e.g., ‘Ferobri’, ‘Guillaume’) and younger cherry cultivars from Dresden–Pillnitz, Germany (e.g., ‘Narana’, ‘Naprumi’), mostly descending from the French variety ‘Souvenir des Charmes’. Sub.III and Sub.IV included 15 cultivars that mainly originated from countries outside Germany, for example ‘Bing’ from USA, ‘Merton Late’ and ‘Merchant’ from Great Britain, or ‘Große Prinzessin’ from The Netherlands. Additionally, several German cherry cultivars, distributed mainly (but not exclusively) in the southern and northern part of Germany, were grouped in these clusters.

### 2.4. Parentage Analysis

Parentage analysis was performed for 56 cherry cultivars with known parents and an existing genetic data set of at least one parent in order to verify ancestral information from the literature (Table 3).

For 31 cherry cultivars, both potential parents known from the literature were genetically investigated in this study, therefore genetic data of both parents were available. For these cultivars, the both-parents–progeny trio confidence was calculated using CERVUS. As result, for all cherry cultivars, the parental information was confirmed based on either a strict confidence of 95% (4 cultivars) or a loose confidence of 80% (26 cultivars). Uniquely, for the cherry cultivar ‘Nafrina’, the parents–progeny trio confidence was <80%, indicating that one of the parents could be wrong.

For 18 cherry cultivars, only the potential mother was genetically tested in this study. For these cultivars, the mother–progeny pair confidence was calculated using CERVUS. As a result, the putative mother was confirmed for all cherry cultivars. For 14 out of 18 investigated cultivars, confidence over 95% was determined for the presumed mother. Uniquely, for the cultivar ‘Katalin’, the mother–progeny pair confidence was below 80%, so it can be assumed that the presumed mother was erroneous.

For seven cultivars, only genetic data of the potential father was available. For these cultivars, the father–progeny pair confidence was calculated using CERVUS. As result, the putative father could be confirmed for six of seven cultivars with a confidence >95%. One exception was the cultivar ‘Swing’, for which ‘Stella’ may not be the true father.

## 3. Discussion

The German Fruit Genebank, as a national decentralized genebank network for fruit genetic resources, was established to ensure the effective, long-term conservation and availability of fruit genetic resources for research and breeding. These genebank collections contain mainly German cultivars, including new German selections and cultivars with sociocultural, local, or historical relation to Germany. However, the collections also contain foreign and new cultivars with important pomological traits, especially for breeders. Currently, sweet cherry genetic resources are maintained by eleven partners. In this study, 1442 trees were examined from six collections that had joined the German Fruit Genebank at the time of this study. Several cultivars were present at more than one collection site; these cultivars were sampled multiple times and used for pomological evaluation and SSR genetic analysis in order to identify cultivar authenticity. The aim of combining pomological and molecular determinations was to analyze the trueness-to-type of the cherry cultivars as accurately as possible. Comparison of the results of both determination methods allowed a better identification of potential errors. Especially in the case of cultivars that were difficult to identify, a more accurate assessment of cultivar authenticity could be made combining pomological and molecular determinations than if only one of these methods were used. Beyond the actual comparison of pomology–molecular genetics, the molecular results allowed further investigation into the relationship of the cultivars.

### 3.1. Pomological Differentiation of Cultivars

The pomological evaluation of cherry cultivars in this study has shown that the determination of the trueness-to-type of cultivars in old collections must necessarily be verified, since numerous accessions were labeled with an incorrect cultivar name. Furthermore, there are numerous synonyms that must be assigned to key names to avoid duplication and to keep the cultivar descriptions in the database correct. A prerequisite for pomological verification is the clear expression of distinctive phenotypic traits that allow the identification of the cultivar by pomologists. Reference cultivars in other collections, stone references, and reference literature are essential for pomological classification. If cultivars are inconsistent in phenotypic characteristics, cultivar authenticity may remain unconfirmed, as was the case with several cultivars in this study. For this reason, genetic analysis with molecular markers is an additional useful tool for evaluating cultivar authenticity [13,14,15,16].

The high identification rates of this study resulted from extensive, critically evaluative reference comparisons with historical stone samples, reference fruit samples and intensive evaluation of historical pomological literature (Table 1). For cultivars originating from other genebank collections, the cultivar names were usually better documented than in orchards (*on farm*), where information about the cultivars planted decades ago were usually lacking. The cultivars originating from the landscape, more often, could not be assigned to a pomologically-described cultivar due to missing references. These cultivars were given provisional working names (marked with ‘An’ for ‘assumed name’ after the cultivar name, [10]). Often, these were cultivars with greater regional or even supra-regional importance.

### 3.2. Choice of Markers

Many genetic studies on cherry demonstrated the usefulness of reliable polymorphic SSR markers for determination of the trueness-to-type and the analysis of genetic diversity and structure in genetic resources [14,17,18,19]. For a better comparability of genetic data with other *Prunus* studies and a future usage of a joint international cherry SSR marker database, we used a set of 16 SSR markers recommended by the ECPGR *Prunus* working group [10,20]. All 16 SSR markers used in our study showed clear polymorphisms and reproducible results with one or two amplified fragments per genotype. For most SSR markers, the power of discrimination was high and combinations of only six markers with the lowest *PI*-values were necessary to discriminate all genotypes in our entire cherry collection (Figure 2). This result was comparable to other genetic studies on *Prunus avium* using SSRs [21,22] and the values of several genetic diversity parameters (e.g., *He*, *Ho* and *PIC*) were considerably higher than using SNP markers for sweet cherry cultivar discrimination [3]. However, the simple *PI* calculation was probably inaccurate, because it did not consider a possible close relationship between the cherry cultivars [23]. Therefore, we also calculated the *PI_sib_* value, which takes into account the existence of related cultivars in the cherry collection. Using this equation, the number of markers needed for the individual discrimination increased to 14, which still confirmed that the number of markers used in this study was sufficient.

### 3.3. Genetic Differentiation of Cultivars in the German Cherry Collection

In our study, it was possible to identify 383 unique genotypes in the six investigated sweet cherry collections. Several accessions with the same name from the different collections were confirmed as duplicates, as their SSR profiles were identical. With support of their phenotypic characteristics, about 86% of the investigated trees were assigned to cultivar names with certainty (Table 1). A similar high genetic level of redundancy between accessions was also observed within other fruit germplasms, supporting their identification as an essential step before estimating genetic diversity and structure of the germplasm [14,15,24,25,26]. The remaining 14% of the trees in our study could not be clearly assigned to a cultivar name. This was the case, for example, if a cultivar name could be assigned to a unique genotype, but no pomological reference was available.

These ambiguities and mistakes underline the need for detailed analysis. Compared to the other collection institutions involved in the study, at the JKI and the Hessian State Office for Agriculture, a slightly higher proportion of cultivars with “own molecular group but without references for pomological variety identification” was found, probably due to the newer international cultivars in these collections.

In our study, for the evaluation of cultivar identity based on genetic data, the threshold for true-to-type was set at >90% allele pattern similarity. This threshold took into account a non-negligible level of PCR artifacts during SSR analysis, which generally occur at a rate of approximately 10% [27,28]. However, allowing for such a margin of error accepted that individual cultivars were considered identical and authentic, when in fact they were different [29]. On the other hand, in our cherry collection, even accessions with 100% identical allelic pattern, such as ‘Querfurter Königskirsche’ and ‘Büttners Rote Knorpelkirsche’ were likely to be false duplications as these accessions were differentiated into two cultivars based on few phenotypic characteristics. These accessions could be synonyms with a common genetic origin that differ only in a few phenotypic traits. Similar observations were reported by Cipriani, et al. [30] after SSR analysis in grapevine. In this study, many grapevine accessions had identical allele patterns but showed different phenotypic traits, explained by genetic mutations. This example demonstrated the importance of both pomological evaluation and genetic analysis for reliable cultivar evaluation. Cherry cultivars with questionable or missing names must be subjected to further pomological evaluation and matched with passport data to clarify the identity of these cultivars. Also of particular importance is the linking of cultivars to historical references by pomologists.

### 3.4. Diversity Parameters, Genetic Structure, Geographic Origin

In our study, the 383 unique genotypes identified in the sweet cherry collections were investigated regarding genetic diversity and structure. As expected for self-incompatible cultivars, genetic diversity in the collection was high. However, compared to other self-incompatible cultivars, such as apple or pear, the genetic diversity was lower in sweet cherry [31,32,33]. In our study, we found that some samples differed from each other at only a few loci. It is possible that the lower genetic diversity could be explained by the frequent occurrence of siblings in the cherry collection. Nevertheless, the genetic diversity in the sweet cherry collection was in accordance with other studies on genetic diversity in cherry [17,18,19,34,35].

After STRUCTURE analysis, the 383 genotypes/cultivars were grouped into three genetic clusters (Figure 3). The low degree of genetic admixture of genotypes in their respective cluster indicated a distinct genetic structure within the German cherry cultivars. This result was contrary to a study of the genetic structure of sweet cherry cultivars from 19 European countries, in which 89% of the genotypes showed a genetic admixture [35]. This could be explained by the fact that only 13 German cultivars were represented in the European study, which may have underestimated the genetic structure of German cherry cultivars. Despite the presence of distinct STRUCTURE clusters in our study, no clear relationship between genetic structure and geographic origin, year of origin, or other phenotypic traits was found. Both older cultivars and younger cherry cultivars, as well as cultivars from Germany and foreign cultivars, were mixed in the three genetic clusters. This demonstrated the extensive involvement of numerous old and foreign cultivars in the development of cherry cultivars by selection after open pollination or breeding work over time [30]. The number of clusters after STRUCTURE analysis was in accordance with the phylogenetic tree drawn by DARwin, which also separated the accessions into three main clusters (Figure 4). However, comparing with STRUCTURE, the phylogenetic tree better reflected the origin of selected cherry cultivars. For example, one branch of the phylogenetic tree combined mostly foreign cherry cultivars in which the main part originated from the Canadian Research station at Summerland, such as ‘Van’, ‘Stella’ or ‘Lapins’. Selected Canadian cultivars from the first breeding release, such as ‘Van’ and ‘Stella’, originating from European, and especially German, cultivars from the Summerland research station were closely related to German cherry cultivars. Similar observations were made for German cultivars that originated from the Fruit Research Station Jork in Germany. A main part of the standard cultivars from the Jork breeding program, such as ‘Erika’, ‘Johanna’, ‘Valeska’, ‘Oktavia’, ‘Regina’, and ‘Karina’, released in the 1950s, were grouped into one branch [36]. All these cultivars are descendants of the sweet cherry cultivar ‘Rube’, which was also included in this branch. Single cherry cultivars of the breeding program of the Institute of Breeding Research on Fruits in Dresden–Pillnitz were combined together with selected French cultivars into one branch. This also reflected the relationship with ancestral cultivars, since ‘Navon’ and ‘Narana’ are descendants of ‘Souvenir des Charmes’.

However, these relationships did not document the geographic origin of older cherry cultivars. In our study, we could not find a clear relationship between the geographic distribution of old cherry cultivars and their genetic structure. This reflected the traditional exchange of plant material across geographic regions and the subsequent genetic admixture during the development of newer cultivars over time [24]. Exceptions were observed for some cultivars from the middle (Harz, Kyffhäuser Region, Werder) and eastern parts (Guben) of Germany, which were combined into one cluster. The middle German regions (including Harz, Kyffhäuser and Werder) are historically important cherry growing areas that originated several old cultivars, such as ‘Werdersche Frühe’ or ‘Kassins Frühe’. Guben also has a long fruit breeding and growing tradition but represents a smaller, limited cultivation area. This area was particularly suitable for cherry cultivation due to local conditions, and supplied the city of Berlin. Several old cherry cultivars of the 19^th^ century originated from random seedling selections in this region, e.g., ‘Schneiders Späte Knorpelkirsche’, ‘Große Germersdorfer’, ‘Dönissens Gelbe’, or ‘Fromms Herzkirsche’. However, for several cultivars, it was not possible to make a definite statement about the geographical origin, as the origin was not always documented.

### 3.5. Parentage Analysis

The parentage analysis in our collection was performed only for cultivars with at least one genetically investigated parent (Table 3). In our study, for 55 of 58 cultivars, the parentage was correctly indicated in the literature or old records. For the remaining cultivars, it was possible that wrong parents were specified. This frequency of incorrect pedigree information was remarkably low, compared to a large-scale parentage study on *Vitis* [37]. In the *Vitis* study, much pedigree information was incorrect. The main reason for these errors was thought to be pollen contamination during hand pollination [37]. That concern extends also to younger cultivars originating from fruit breeding programs, which usually use controlled hand pollination. Pollen contamination could be the reason for the misclassification of the parentage of the one cultivar in our study, as the father was possibly classified incorrectly.

Incorrect cultivar identification or synonymy in parents’ names is obviously a second involuntary reason that can lead to pedigree errors [37]. This is an especially likely explanation if the mother is not correct. Of note, for older cherry cultivars, numerous synonyms exist. For example, in the past, the two parent cultivars ‘Werdersche Frühe’ and ‘Büttners Rote Knorpelkirsche’ were often confused with other cultivars in cherry collections, which could explain the possible misclassification of one parent of ‘Nafrina’, since at least 16 synonyms exist for both parents (Deutsche Genbank Obst, DGO; https://www.deutsche-genbank-obst.de/, Cherry Network, accessed on 1 December 2022).

In our collection, the confidence level of confirmation was significantly lower for the varieties with two known parents than for those with only one known parent. One reason for this could be erroneous parent assignment due to genotypes containing typing errors. Particularly in collections with a close genetic relationship, as between the investigated sweet cherry genotypes in our study, the assignment of true cultivars can be challenging. Allowing typing errors may improve assignment, but it can impede a clear distinctive parent identification in the collection. Another reason may be more closely related to the analysis itself, since the LOD value for assessing true inheritance is lower when the potential parents carry predominantly common alleles than when they carry more rare alleles. The absence of rare alleles in the cherry collection in our study could explain the low LOD value (confidence level < 95%) for some of the parents studied (data not shown).

Therefore, parentage analysis should generally be considered with caution, since these results can be very speculative. Reliable results can only be discovered through extensive genotyping of large collections and additional observations of pomological traits [30,38].

## 4. Materials and Methods

### 4.1. Plant Material

**Figure 5:**
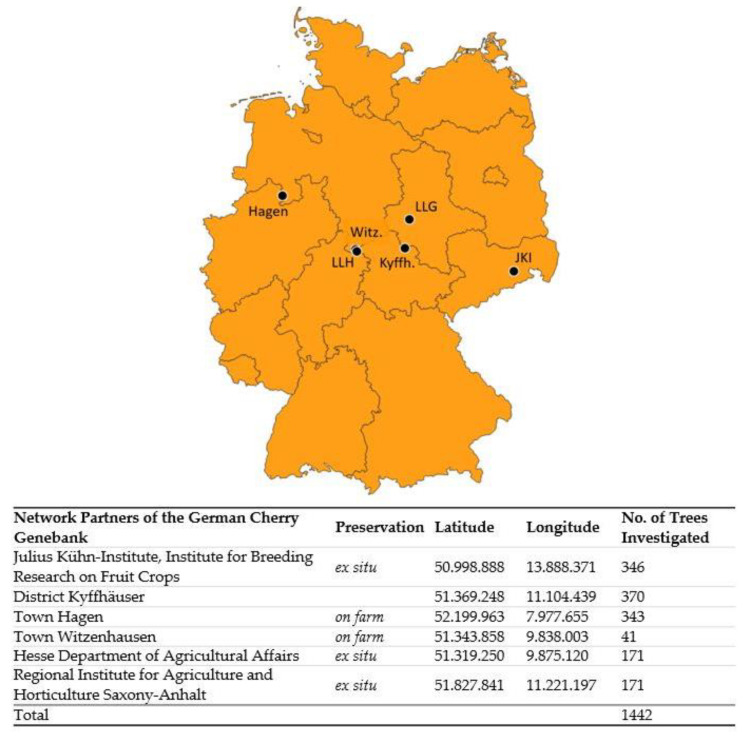
Cherry network collections of sweet cherry of the German Fruit Genebank investigated. Geographic location and the number of trees evaluated for trueness-to-type, preserved at six different locations in Germany. Hagen: Town Hagen a. T. W.; JKI: Julius Kühn-Institute, Institute for Breeding Research on Fruit Crops, location Dresden–Pillnitz; Kyffh.: District Kyffhäuser, location Bad Frankenhausen; LLH: Hesse Department of Agricultural Affairs, location Witzenhausen–Wenderhausen; LLG: Regional Institute for Agriculture and Horticulture Saxony–Anhalt, location Ditfurt; Witz.: Town Witzenhausen.

On behalf of the German Federal Ministry of Food and Agriculture, four projects were conducted from 2009 to 2014 and from 2017 to 2020 to characterize the trueness–to-type of the cultivars in the cherry network of the German Fruit Genebank. A total of 1442 sweet cherry (*Prunus avium* L.) individual trees, preserved by six different genebank network partners, were investigated (Figure 5). The collections were managed as ex situ field collections and *on farm*, mostly grown on seedling rootstocks in landscaped orchards (*on farm* conservation). The cultivars within the cherry network of the German Fruit Genebank included (1) German cultivars, including new German selections, (2) cultivars with sociocultural, local or historical relation to Germany, and (3) cultivars with important pomological characteristics, especially for breeders. Some cultivars were present in several collection sites and could be sampled multiple times. The characterization of cultivar identity was based on two evaluation procedures: the pomological evaluation and the molecular analysis.

### 4.2. Pomological Characterization

One main topic addressed was the characterization based on pomological traits by two members of the Pomological Commission of the German Pomological Society. The pomological identification was performed during natural fruit ripening in the years 2009 to 2011 and repeated in the years 2017 to 2020. In the second evaluation, the trees were analyzed, for which the first characterization showed ambiguities or which had no fruits. In addition, new trees from new plantings were also included in the assessments. The pomological identification was based on qualitative comparison of cultivar-descriptive phenotypic traits, which mainly included various fruit, tree, and especially stone characteristics [6]. Important fruit traits included time of ripening, fruit color, shape (side and ventral view), relief (characteristics of pistil and stem pit), flesh color, firmness and flavor, and fruit stem characteristics. The expression of stone traits were of particular importance, including the stone form (side and ventral view) and characteristics of the grooves and ridges of the ventral bulge. For the determination of fruit characteristics, 15–20 large and well-formed fruits per tree were used [6]. Once the fruit sample review was completed, another review step was performed based solely on the archived fruit stones. The identification was carried out using multiple reference sources, such as fruit references from historical cherry variety collections and stone references from historical stone collections of the Federal Plant Variety Office. In addition, extensive literature evaluation of historical pomological sources was included.

Tree, fruit and stone photos were taken for each cultivar and uploaded to the database of the German National Fruit Genebank (Deutsche Genbank Obst, DGO (https://www.deutsche-genbank-obst.de/, Cherry Network).

### 4.3. DNA Extraction and Genetic Analysis

The second evaluation procedure was the molecular DNA fingerprint analysis, which was realized in the years 2012 to 2014 and 2017 to 2019. The microsatellite primers were selected according to the guidelines of the ECPGR *Prunus* Working Group, which recommended a standard set of 16 SSR markers [10]: BPPCT037 [39], CPPCT006, and 022 [40], EMPA002, 003, 017, and 026 [10], EMPaS01, 02, 06, 10, 12, and 14 [22], PceGA34 [41], PS05C03 [42], and UDP98-412 [43] (Table S1). These markers were developed mainly from *P. avium* and provided good coverage of the cherry genome (two unlinked markers per linkage group).

The first sample set, collected in 2012–2014, was genotyped by the Competence Centre for Fruit Production–Lake Constance (KOB, Ravensburg, Germany). Leaf material of the single trees was collected from the different collection sites in 2 mL reaction tubes and dried using silica gel according to a modified protocol by Slotta, et al. [44]. The leaf material was stored at room temperature until DNA isolation. The DNA isolation was performed using the DNeasy Plant Mini Kit (Qiagen, Hilden, Germany) according to the manufacturer’s instructions. Multiplex PCR reactions were carried out using the Taq DNA Polymerase (cloned) (GE Healthcare Life Science, Freiburg, Germany) according to the manufacturer’s instructions, with an initial denaturation at 94 °C for 2 min, followed by 37 cycles of denaturation at 94 °C for 45 s, annealing at 56 °C for 1 min, and elongation at 72 °C for 2 min. The PCR reaction was completed with a final elongation step at 72 °C for 10 min. Fragment lengths analysis was done on a CEQ 8000 capillary sequencer (Beckman Coulter GmbH, Krefeld, Germany) using the GenomeLab™ GeXP software (Beckman Coulter GmbH, Krefeld, Germany).

The second sample set, collected in 2017–2019, was genotyped by Ecogenics GmbH c/o Microsynth AG (Balgach, Switzerland). Leaf material of each single tree was collected from the different collection sites in a sample bag and was immediately stored on dry ice. Until DNA isolation, the samples were stored at −80 °C. For DNA isolation, the Hotshot method, according to Truett, et al. [45], was used. In order to remove any PCR inhibitors, an additional DNA purification step was performed using the OneStep PCR Inhibitor Removal Kit (Zymo Research, Freiburg, Germany). Multiplex PCR was performed using the Type-It kit (Qiagen, Germany) according to the manufacturer’s instructions, with an initial denaturation at 95 °C for 2 min, followed by 40 cycles of denaturation at 94 °C for 0.5 min, annealing at 55 °C (48 °C for EMPaS01 und PS05C03) for 1.5 min, and elongation at 72 °C for 1 min. The PCR reaction was completed with a final elongation step at 72 °C for 30 min.

Fragment lengths analysis was done on a 3730XL DNA-Analyzer (Applied Biosystems, Waltham, MA, USA) using the Software GeneMarker V2.6.4 (SoftGenetics LLC., State College, PA, USA). In addition, the fragments were visually checked for appropriate quality. The eight genotypes *P. avium* F12/1, *P. avium* ‘Goodnestone Black’, *P. avium* ‘Napoleon’, *P. avium* ‘Noble’, *P. avium* ‘Noir de Meched’, *P. incisa* E621, *P. mahaleb* SL64 and *P. nipponica* F1292 (also recommended by the ECPGR), were used as reference cherry genotypes [10]. These reference genotypes allowed the harmonization of the SSR fingerprints originating from the two different laboratories that used different fragment length analysis protocols and devices for the genotyping.

### 4.4. Probability of Identity (PI) Calculation

To estimate the number of marker combinations required to distinguish all genotypes, the identity probability (*PI*) was calculated using GenAlex ver 6.5. In estimating *PI*, it was assumed that the individuals being compared were unrelated. Because the samples could have contained relatives, we also calculated *PI_sib_*, which gave a more conservative estimate of *PI*.

The lower the value for *PI*, the higher the probability that the marker could distinguish genotypes in the entire data set.

### 4.5. Assessment on Cultivar ‘Trueness-to-Type’

**Figure 6:**
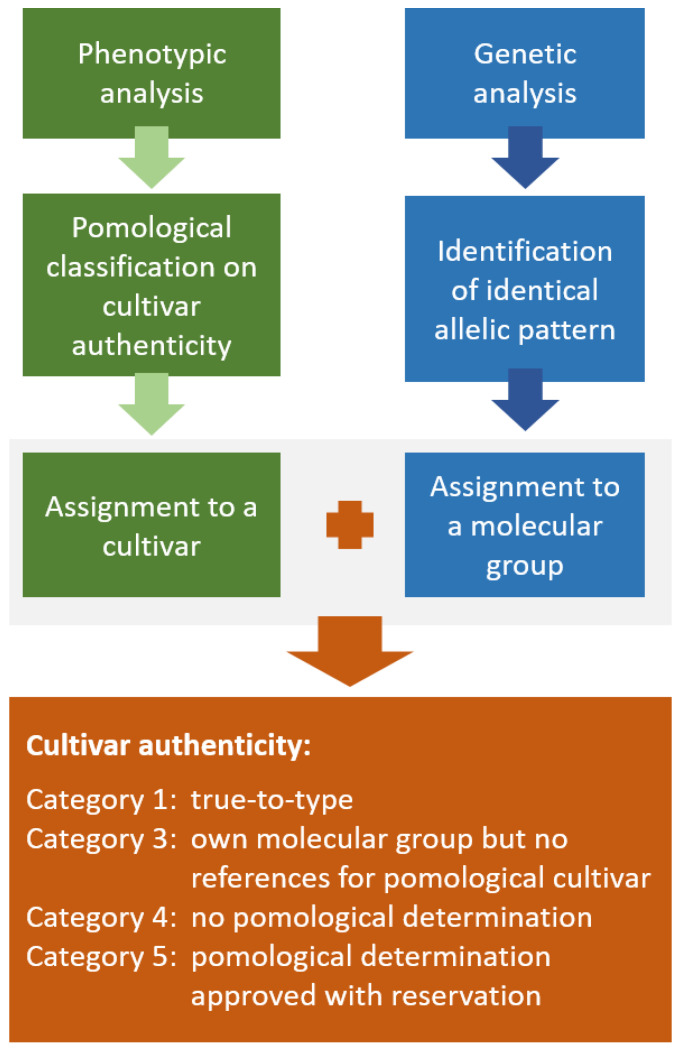
Workflow for the assessment of cultivar authenticity in sweet cherries based on phenotypic and genetic data (category 2 is missing because it is only used for categorizing sour cherries).

In general, accessions with the same or a synonymous cultivar name were assumed to be true-to-type if they had identical marker profiles. Identity analysis was performed using the software CERVUS 3.0.7 [46,47]. For this analysis, the data from the first project were calculated together with the results of the second analysis. Similarities between the plants were calculated using a simple matching coefficient. Small allele size differences of ±1 bp were not considered, as they are often due to technical uncertainties. Furthermore, it was taken into account that any PCR analysis could have some margin of error, resulting in single additional or missing fragments [27]. Therefore, the threshold for assessing whether the accessions were identical was set at 90% similarity. All accessions with similar allelic pattern >90% were considered identical and included in one molecular group. The genotype most frequently observed within a group was assumed to represent the cultivar, while accessions with the same names but different allele patterns were considered inauthentic and falsely labeled. At the same time, almost all sweet cherry cultivars were evaluated pomologically to confirm or reject the assumed cultivar identity. Finally, the pomological and genetic affiliation were compared to eventually confirm the authenticity of the cultivar (confirmation of the original assigned cultivar name or assignment of the new determined name, as shown in Figure 6, category 1) or conditionally approve the pomological determination (Figure 6, category 5).

### 4.6. Diversity Parameter, STRUCTURE Analysis and Phylogenetic Tree Construction

The mean number of alleles by locus (*Na*), effective number of alleles (*Ne*), observed heterozygosity (*Ho*) and expected heterozygosity (*He*) were calculated for each nuclear SSR loci within the 383 cherry cultivars using the software GENALEX ver. 6.5 [48,49]. Allelic richness (*Ar*) was calculated with the software ADZE [50] using the rarefaction method to correct differences in the genetic data set because of missing data [51].

Genetic structure within the 383 unique genotypes of the entire cherry collection was analyzed using the software program STRUCTURE. The parameters were 100,000 burn-in periods and 100,000 Markov Chain Monte Carlo repetitions using the admixture model with correlated allele models. To estimate the number of genetic groups in the cherry collection, we ran the program from 2 to 8 with 5 runs for each *K* value. STRUCTURE HARVESTER [52] was used for detecting the most likely value for *K* based on Evanno’s ΔK method [53]. The genetic differentiation between the single genetic clusters was measured by Wrights fixation index (*F_st_*) [12] using GENALEX ver. 6.5.

The program DARwin ver.5 was used to estimate the dissimilarity distance matrix of the genetic data of the 383 genotypes considering a bootstrap analysis with 1000 replications [54]. The tree was constructed with DARwin ver.5 using the unweighted neighbor joining method [55]. The tree figure was generated using Dendroscope ver.2.7.4 [56].

### 4.7. Parentage Analysis

CERVUS software version 3.0.7 [46,47] was used to verify the parentage of 56 cultivars with an existing genetic data set of at least one parent. Genetic data were available for the potential father only (seven cultivars), the potential mother only (18 cultivars), or both potential parents (31 cultivars), respectively. For parent, maternity, and paternity analysis, the following parameters were considered: progeny simulated 100,000; proportion of candidate parents sampled = 0.95; proportion of loci typed = 0.9; proportion of loci mistyped = 0.1; minimum number of typed loci = 13; relaxed confidence level = 80%, strict confidence level = 95%. Each analysis completed an allele frequency analysis, followed by a simulation of parent, maternity, and paternity analysis wherein the number of potential father and mothers each were set to 31, 18, and 7, respectively. Based on these parentage simulations, the respective LOD values were calculated to determine the confidence levels for the assignment of the most likely parents (trio confidence), or most likely fathers and mothers (pair confidence) for each genotype.

## 5. Conclusions

Pomological and genetic characterization of cultivars in genebank collections is an essential prerequisite for confirming the trueness–to-type of cultivars and genebank management. In our study, the trueness-to-type of 86% of cherry trees could be confirmed based on pomological and genetic evaluation. For the remaining unauthenticated accessions, additional pomological studies are needed to clarify open questions on authenticity. In general, a high degree of genetic admixture was found according to geographical location and year of origin, demonstrating extensive exchange of genetic information between old and younger cultivars and between German and foreign cultivars over time. A single parentage could not be confirmed, indicating the need for further pedigree analyses for this cultivar. The results of our study underlined the necessity of assessment of true-to-type cultivars in genebank collections based on genetic and pomological evaluation.

The results of the sweet cherry pomological and molecular analyses were forwarded to the DGO partners of the German National Fruit Genebank for revision of their collections and updating of the database (https://www.deutsche-genbank-obst.de/). With the determination of cultivar authenticity, the goal of the conservation strategy of the German National Fruit Genebank to secure each variety in at least two different locations can be achieved. Thus, the varieties confirmed as authentic can now be exchanged between the collection partners.

## Figures and Tables

**Table 1:** Determination of true-to-type cultivars based on pomological and molecular analyses of sweet cherry trees from the German Fruit Genebank (six partners involved).

Result of Assessment	No. Trees	% Trees
True-to-type	1235	85.64
Own molecular group but no references for pomological cultivar identification	120	8.32
No pomological determination	64	4.4
Pomological determination approved with reservation	23	1.6
Total	1442	

**Table 2:** Genetic parameters for the 16 SSRs used in the molecular analysis of the sweet accessions of the German National Fruit Genebank.

Locus	*N*	*Na*	*Ne*	*Ho*	*He*	*Ar*
BPPCT037	383	15.00	4.87	0.83	0.79	9.51
CPPCT006	383	15.00	4.17	0.78	0.76	9.56
CPPCT022	383	14.00	3.56	0.75	0.72	9.89
EMPA002	383	11.00	2.07	0.61	0.52	9.89
EMPA003	383	5.00	1.62	0.40	0.38	3.11
EMPA017	383	13.00	1.41	0.27	0.29	6.94
EMPA026	382	14.00	2.20	0.51	0.55	8.02
EMPaS01	383	11.00	3.13	0.71	0.68	11.89
EMPaS02	383	15.00	3.71	0.75	0.73	10.55
EMPaS06	383	23.00	7.43	0.86	0.87	15.56
EMPaS10	383	20.00	3.09	0.61	0.68	11.71
EMPaS12	383	11.00	4.27	0.77	0.77	12.89
EMPaS14	383	9.00	2.43	0.61	0.59	10.89
PceGA34	256	24.00	7.47	0.88	0.87	13.89
PS05C03	258	20.00	4.61	0.78	0.78	14.97
UDP98-412	383	23.00	5.08	0.62	0.80	16.27
Mean		15.19	3.82	0.67	0.67	10.97

*N =* number of samples; *Na*: number of different alleles; *Ne*: number of effective alleles (=1/(∑ *p_i_*^2^)); *p_i_*: relative frequency of the ith allele; *Ho*: observed heterozygosity (=number of heterozygotes/N); *He*: expected heterozygosity (=1 − ∑ *p_i_*^2^); *Ar* = Allelic richness.

**Table 3:** Parental analysis for 56 sweet cherry cultivars of the German Fruit Genebank collection, with known parents and an existing SSR genetic data set of at least one parent.

Cultivar Name	Assumed Mother	Assumed Father	Trio Confidence ^1^
Alma	Rube	Allers Späte	+
Annabella	Rube	Allers Späte Knorpel	+
Areko	Kordia	Regina	+
Bianca	Rube	Allers Späte	+
Erika	Rube	Stechmanns Bunte	+
Ferbolus	Hedelfinger Riesenkirsche	Reverchon	+
Ferobri	Burlat	Fercer	+
Glacier	Stella	Burlat	+
Habunt	Valeska	Sunburst	+
Johanna	Schneiders Späte Knorpelkirsche	Rube	+
Karina	Schneiders Späte Knorpelkirsche	Rube	+
Lapins	Van	Stella	+
Merton Bounty	Elton	Schreckenskirsche	*
Müncheberger Fruehernte	Flamentiner	Früheste der Mark	+
Nafrina	Werdersche Frühe	Büttners Rote Knorpelkirsche	-
Namada	Badeborner Schwarze Knorpelkirsche	Rivers Frühe	+
New Star	Van	Stella	+
Oktavia	Schneiders Späte Knorpelkirsche	Rube	+
Rainier	Bing	Van	+
Regina	Schneiders Späte Knorpelkirsche	Rube	+
Ria	Kordia	Vic	+
Somerset	Van	Vic	+
Summit	Van	Sam	+
Sunburst	Van	Stella	+
Sylvia	Van	Sam; Van	+
Techlovan	Van	Kordia	*
Valeska	Rube	Stechmanns Bunte	+
Vanda	Van	Kordia	*
Vega	Bing	Victor	*
Viola	Schneiders Späte Knorpelkirsche	Rube	+
Vista	Hedelfinger Riesenkirsche	Victor	+
**Cultivar name**	**Assumed Father ^#^**	**Assumed Mother**	**Pair confidence ^2^**
Beata	open pollinated	Lambert	*
Chelan	Beaulieu	Stella	*
Chinook	Gil Peck	Bing	*
Ferprime	open pollinated	Fercer	*
Katalin	Podjebrad sarga	Schneiders Späte Knorpelkirsche	-
Lambert	Black Heart	Große Prinzessin	*
Lamida	open pollinated	Lambert	+
Larian	UCD 50 (Bing x Bush Tartarian)	Lambert	*
Linda	Germersdorfer	Hedelfinger	*
Magda	open pollinated	Basler Adlerkirsche	*
Merchant	open pollinated	Merton Glory	+
Merla	open pollinated	Merton Late	*
Mermat	open pollinated	Merton Glory	*
Merpet	open pollinated	Merton Glory	*
Nalitta	open pollinated	Querfurter Königskirsche	*
Sodus	Giant	Große Prinzessin	*
Stella	JI 2420 (Emperor Francis x Napoleon X-rayed pollen)	Lambert	*
Vic	Schmidt	Bing	+
**Cultivar name**	**Assumed Mother ^#^**	**Assumed Father**	**Pair confidence ^3^**
Bing	Black Republican	Große Prinzessin	*
Fernier	Tardif de Vignola	Rainier	*
Merton Favourite	Knight’s Early Black	Schreckenskirsche	*
Merton Glory	Ursula Rivers	Noble	*
Merton Late	Hildesheim (Belle Agathe)	Große Prinzessin	*
Sandra Rose	2C-61-18 (Star x Van)	Sunburst	*
Swing	Nabigos	Stella	-

^#^ not genetically investigated in this study; ^1^ mother-father–progeny confidence; ^2^ mother–progeny confidence; ^3^ father–progeny confidence; * Confidence 95%; + confidence 80%; - confidence < 80%

## Data Availability

The datasets presented in this study can be found in online repositories [11]. The names of the repository/repositories and accession number(s) can be found in the article.

## Supplementary Materials

The following supporting information can be downloaded at: https://www.mdpi.com/article/10.3390/plants12010205/s1, Table S1: SSR markers used for the genetic assessment of the authenticity of Sweet Cherry Cultivars in the German National Fruit Genebank. Figure S1: Dendrogram of the cherry cultivars investigated in this study.
