# Supplementary material for: Genetic and Pomological Determination of the Trueness-to-Type of Sweet Cherry Cultivars in the German National Fruit Genebank
Source: Plants (Basel). 2023 Jan 3;12(1):205. doi: 10.3390/plants12010205 (PMC9823723; doi:10.3390/plants12010205)
Supplement: Supplementary file 1 [file plants-12-00205-s001.zip › Figure S1_Dendrogram_Cherry_cultivars.pdf]

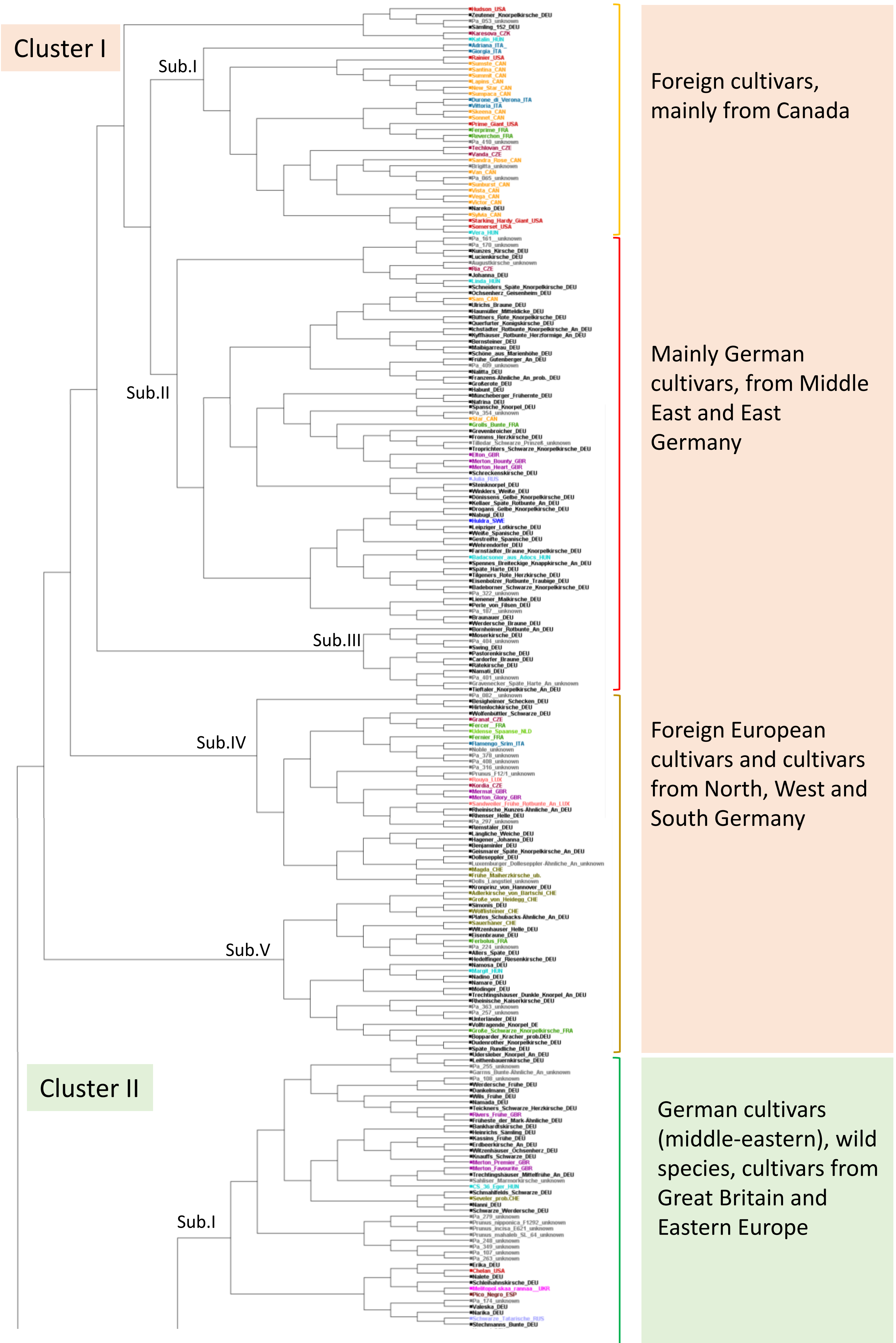
Cluster I
Sub.I
Sub.II
Sub.III
Sub.IV
Sub.V
Cluster II
Sub.I
Foreign cultivars, mainly from Canada
Mainly German cultivars, from Middle East and East Germany
Foreign European cultivars and cultivars from North, West and South Germany
German cultivars (middle-eastern), wild species, cultivars from Great Britain and Eastern Europe

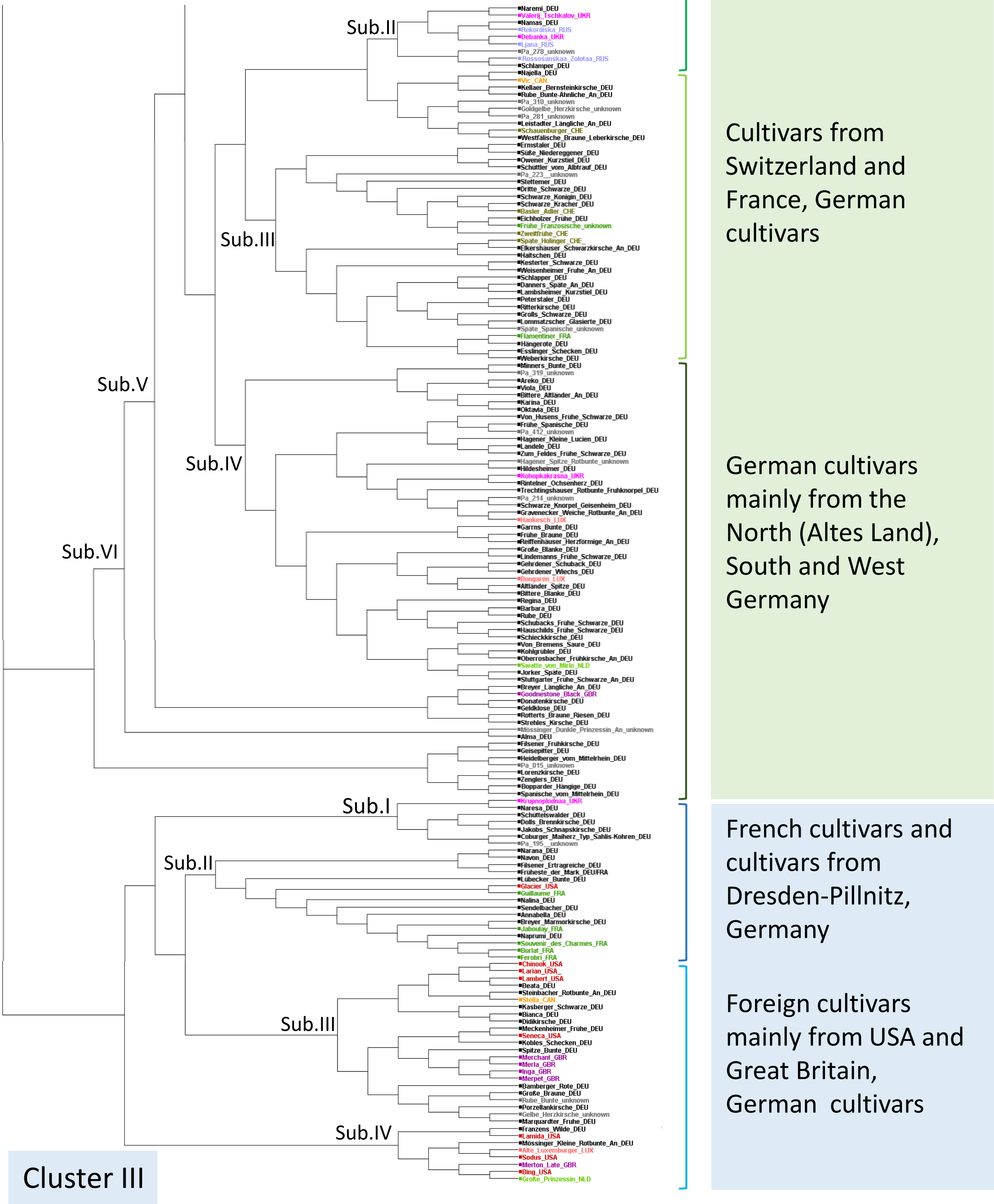
Sub.II
Sub.III
Sub.IV
Sub.V
Sub.VI
Cultivars from Switzerland and France, German cultivars
German cultivars mainly from the North (Altes Land), South and West Germany
Sub.I
Sub.II
Sub.III
Sub.IV
French cultivars and cultivars from Dresden-Pillnitz, Germany
Foreign cultivars mainly from USA and Great Britain, German cultivars
Cluster III
