# Supplementary material for: Genetic and Pomological Determination of the Trueness-to-Type of Sweet Cherry Cultivars in the German National Fruit Genebank
Source: Plants (Basel). 2023 Jan 3;12(1):205. doi: 10.3390/plants12010205 (PMC9823723; doi:10.3390/plants12010205)
Supplement: Supplementary file 1 [file plants-12-00205-s001.zip › Table S1_SSR markers used in this study.pdf]

Table S1. SSR markers used for the genetic assessment of the authenticity of Sweet Cherry Cultivars in the German National Fruit Genebank.

Multiplex	Marker	Primer	Primer sequence (5' → 3')	Dye
MP1	BPPCT037	BPPCT037-F	CATGGAAGAGGATCAAGTGC	FAM
		BPPCT037-R	CTTGAAGGTAGTGCCAAAGC	
	CPPCT022	CPPCT022-F	CAATTAGCTAGAGAGAATTATTG	FAM
		CPPCT022-R	GACAAGAAGCAAGTAGTTTG	
	EMPA002	EMPA002-F	TGACAGGTCATCATACCATTTG	At532
		EMPA002-R	CAGGATTAAGCATTGCAAATTA	
	EMPaS06	EMPaS06-F	AAGCGGAAAGCACAGGTAG	At532
		EMPaS06-R	TTGCTAGCATAGAAAAGAATTGTAG	
	EMPA003	EMPA003-F	AGCCATTCTGAAAAGGTGGA	At550
		EMPA003-R	GCATTCAGCCAACAAAATCA	
	EMPaS14	EMPaS14-F	TCCGCCATATCACAATCAAC	At550
		EMPaS14-R	TTCCACACAAAAACCAATCC	
MP2	CPPCT006	CPPCT006-F	AATTAACTCCAACAGCTCCA	FAM
		CPPCT006-R	ATGGTTGCTTAATTCAATGG	
	UDP98-412	UDP98-412-F	AGGGAAAGTTTCTGCTGCAC	At532
		UDP98-412-R	GCTGAAGACGACGATGATGA	
	EMPaS01	EMPaS01-F	CAAAATCAACAAAATCTAAACC	At532
		EMPaS01-R	CAAGAATCTTCTAGCTCAAACC	
	PS05C03	PS05C03-F	AGATCTCAAAGAAGCTGA	At550
		PS05C03-R	AGCTTATGCATATACCTG	
	EMPA002	EMPA002-F	TGACAGGTCATCATACCATTTG	At565
		EMPA002-R	CAGGATTAAGCATTGCAAATTA	
MP3	EMPaS12	EMPaS12-F	TGTGCTAATGCCAAAAATACC	FAM
		EMPaS12-R	ACATGCATTTCAACCCACTC	
	EMPA017	EMPA017-F	ATTTCAATGTGGGGATGAGC	FAM
		EMPA017-R	TGAAGTGAGGGAAATGGAGC	
	EMPA026	EMPA026-F	ATTGAAAAAGCCAAAGAGCG	At532
		EMPA026-R	TTCACGGTTTGAAGCAAGTG	
	PceGA34	PceGA34-F	GAACATGTGGTGTGCTGGTT	At550
		PceGA34-R	TCCACTAGGAGGTGCAAATG	
	EMPaS10	EMPaS10-F	GCTAATATCAAATCCCAGCTCTC	At565
		EMPaS10-R	TGAAGAAGTATGGCTTCTGTGG	
